# Multi-View Fusion-Based Automated Full-Posture Cattle Body Size Measurement

**DOI:** 10.3390/ani14223190

**Published:** 2024-11-07

**Authors:** Zhihua Wu, Jikai Zhang, Jie Li, Wentao Zhao

**Affiliations:** School of Automation and Electrical Engineering, Inner Mongolia University of Science and Technology, Baotou 014017, China; 2022022396@stu.imust.edu.cn (Z.W.);

**Keywords:** cattle, body measurements, multi-view, point cloud

## Abstract

Cattle farming is an important part of the global livestock industry, and cattle body size is the key indicator of livestock growth. However, traditional manual methods for measuring body sizes are not only time-consuming and labor-intensive but also incur significant costs. Meanwhile, automatic measurement techniques are prone to being affected by environmental conditions and the standing postures of livestock. To overcome these challenges, this study proposes a multi-view fusion-driven automatic measurement system for full-attitude cattle body measurements. Outdoors in natural light, three Zed2 cameras were installed covering different views of the channel. Multiple images, including RGB images, depth images, and point clouds, were automatically acquired from multiple views using the YOLOv8n algorithm. The point clouds from different views undergo multiple denoising to become local point clouds of the cattle body. The local point clouds are coarsely and finely aligned to become a complete point cloud of the cattle body. After detecting the 2D key points on the RGB image created by the YOLOv8x-pose algorithm, the 2D key points are mapped onto the 3D cattle body by combining the internal parameters of the camera and the depth values of the corresponding pixels of the depth map. Based on the mapped 3D key points, the body sizes of cows in different poses are automatically measured, including height, length, abdominal circumference, and chest circumference. In addition, support vector machines and Bézier curves are employed to rectify the missing and deformed circumference body sizes caused by environmental effects. The automatic body measurement system measured the height, length, abdominal circumference, and chest circumference of 47 Huaxi Beef Cattle, a breed native to China, and compared the results with manual measurements. The average relative errors were 2.32%, 2.27%, 3.67%, and 5.22%, respectively, when compared with manual measurements, demonstrating the feasibility and accuracy of the system.

## Simple Summary

Cattle farming occupies an important position in the global livestock industry. The body size of cattle is a major indicator of their growth. The traditional manual measurement of cattle body sizes is both costly and time-consuming, and current automated measurement methods are susceptible to environmental factors. In order to solve these problems, this study introduces a multi-view fusion-driven automated measurement system. In this study, non-contact cattle body size measurement was achieved using multi-view RGB images, depth images, and point clouds. Environmental interference was excluded as much as possible during the process. By using multi-view Zed2 binocular cameras and advanced algorithms, accurate body size measurements were achieved, with small errors compared to manual methods. This demonstrates the potential of the model to improve efficiency and animal welfare in the cattle industry.

## 1. Introduction

In practical livestock management, extreme changes in body measurements of livestock are often caused by disease. Livestock body sizes reflect their growth and development and are an important indicator for assessing their progress. So, it is important to take regular measurements to assess the physical growth of livestock. For the past 50 years, body measurements of livestock have usually been taken manually by surveyors using specialized tools. The accuracy of manual measurements relies on the surveyor’s experience. Regular manual measurements are time-consuming, labor-intensive, and expensive [1]. In recent years, with the rise in non-contact automatic measurement technology, the traditional manual measurement has gradually been withdrawn from use. The animal husbandry industry has been developing rapidly in the direction of automation and artificial intelligence. Many scholars have carried out relevant research [2,3]. Meanwhile, the rapid development of 3D vision sensors provides new opportunities for this transformation. The advantages of these sensors, including high precision, high efficiency, and low cost [4,5,6], are rapidly propelling them into a significant position within the realm of automatic livestock body size measurement. In specific applications, they are mainly classified into single-view measurement and multi-view measurement.

Single-view measurement is generally used to measure livestock body measurements by means of a depth image or point cloud from the specific view. Applying an improved convolutional neural network (CNN) and combining transfer learning and integrated modeling techniques to achieve automatic evaluation of the cow body condition score (BCS) on depth images of the dairy cow’s back [7]. After removing the head and neck from the dorsal depth image of the cow keyframe, an estimation of measurement points based on spatial features is implemented to automatically calculate the body size of the cow [8]. With pigs, a body weight estimation and measurement of local body sizes can be made based on a point cloud of the pig’s back using a convolutional neural network with multiple heads of attention [9]. Many other researchers have used different systems of extracting feature information from single-view images for the automated measurement of livestock bodies [10,11,12,13,14]. However, there are limitations to the single-view angle measurement method. The accuracy of the measurement depends on the standing posture of the animal. The restricted viewing angle makes it difficult to directly measure body circumference, such as the abdominal and chest circumference of livestock.

Due to its abundant feature information, multi-view measurement exhibits greater precision and a broader range in measuring livestock body sizes. Song et al. [15] improved the accuracy of recognizing anatomical markers on the body surface of cattle by extracting more feature information from multi-view depth images. As a result, they achieved high sensitivity in the automated classification of Body Condition Score (BCS) for dairy cattle. Salau et al. [16] measured and analyzed the linear descriptive characteristics of dairy cattle using six Kinect cameras. They successfully acquired key body sizes, including teat length and sciatic node height. Compared with depth images, the highest-dimensional point cloud data can provide complete 3D structural information. It can provide more solid data support for accurate measurement of livestock body sizes. Le et al. [17] set up a Metrux2 camera in multiple views, and scanned livestock to obtain point cloud data to train regression prediction models. These models were then employed to estimate livestock body volume, surface area, and weight. Some other researchers have collected scene point cloud data containing livestock from multiple views. They removed the background and aligned the images into the non-rigid 3D reconstruction. Based on this reconstruction, they accurately measured the livestock’s body sizes [18,19].

The multi-view measurement technique is able to measure livestock body sizes with high spatial complexity, such as body circumference. However, it cannot take into account the effect of the livestock standing posture. In addition, when dealing with point cloud data, environmental factors like occlusion by railing occlusion and uneven natural lighting can cause holes and distortions in the point clouds. This affects the accuracy of the measurements. Setting up a strict measurement environment not only increases the complexity and difficulty of the operation but also significantly increases the time, labor, and equipment costs of the measurement process.

Therefore, the research objective of this paper is to achieve the minimization of the influence of holes and deformation on the measurement accuracy under the natural outdoor complex environment in order to achieve a more accurate measurement of body height, body length, abdominal circumference, chest circumference, and other body measurements of cattle in different postures.

## 2. Materials and Methods

Under outdoor natural light conditions, the YOLOv8n algorithm [20] automatically acquires multi-view image data. After the multi-view point clouds are denoised and registered to form the complete cattle body point cloud, the YOLOv8x-pose algorithm [21] detects 2D key points on the RGB images. These key points are then mapped to the 3D cattle body using the depth map and internal camera parameters, enabling automatic measurement of the cattle’s body sizes across full poses.

**Figure 1:**
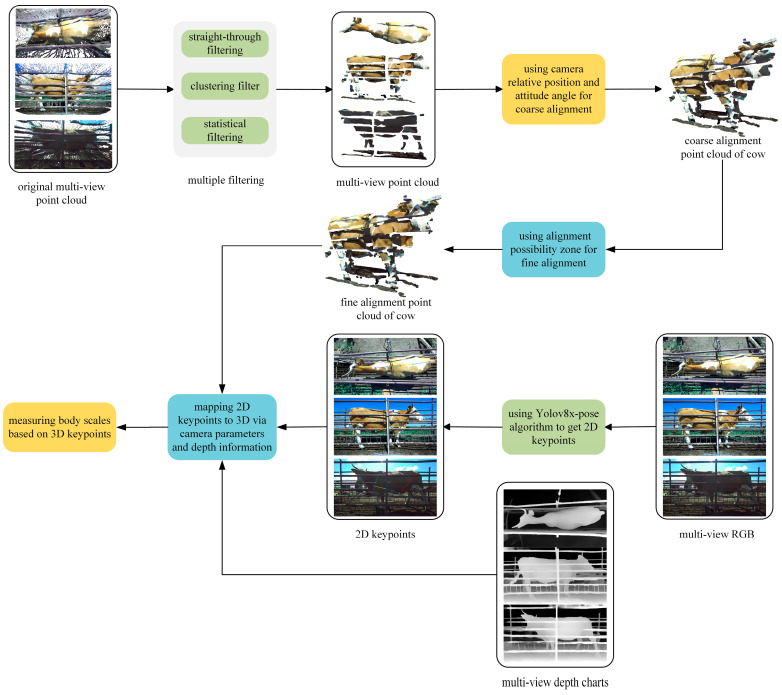
General flow of the automatic measuring device.

The overall process flow of the automatic measurement device is shown in Figure 1.

### 2.1. Automatic Measurement Device

The automatic measuring device in this study uses three Zed2 cameras. It can acquire RGB images and depth images with a maximum resolution of 2208 × 1242 pixels; a 110° diagonal, 70° horizontal, and 120° vertical field of view; and an effective range of 0.3–20 m. It supports 100 frames of data acquisition.

**Figure 2:**
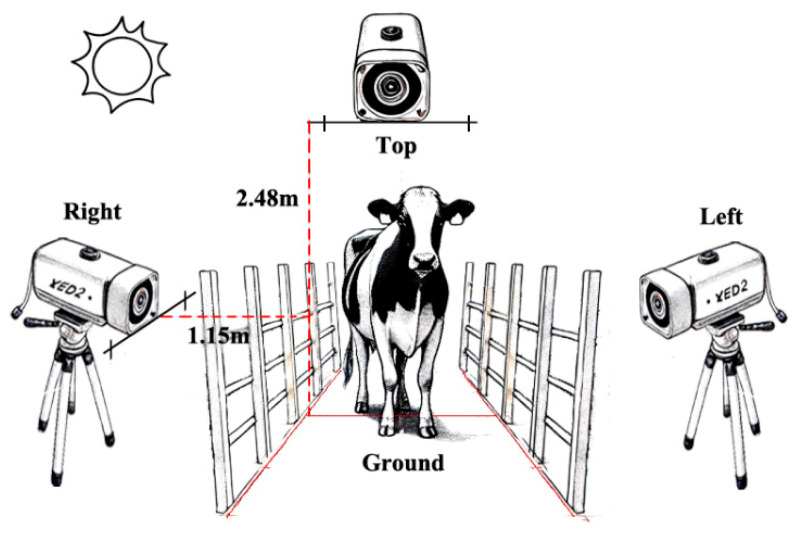
Schematic diagram of the automatic measuring device.

The study adopts the multi-view camera layout strategy, positioning the Zed2 stereo camera on each of the left, right, and top sides of the passage. This arrangement guarantees that the images captured from varying angles complement one another, yielding more comprehensive and precise body posture information [22]. As shown in Figure 2, under natural outdoor lighting conditions, the set up involved positioning the cameras in specific locations. The top camera was mounted 2.48 m above the ground, directly above the channel. The right camera was situated in the sunny area, while the left camera was placed in the shady area. Both the left and right cameras were securely fixed on tripods, and each tripod was positioned 1.15 m away from the channel fence. Each Zed2 camera connects via USB 3.0 to the host PC with the Windows 11 operating system, each with its own independent work thread. The PC hardware configuration is 32 GB of RAM, an Intel I7-12700KF processor, and an NVIDIA 3080 10G graphics card.

In the Wulagai Management Area, Xilingol League, Inner Mongolia, 1200 sets of RGB images of cattle exhibiting various postures, including walking and standing, were collected within the channel, both indoors and outdoors. Each image was marked with the cattle’s body, head, and rump. These annotations formed the deep learning dataset. This dataset was then used to train the YOLOv8 deep learning model specifically designed to detect these precise regions of the cattle within images.

**Figure 3:**
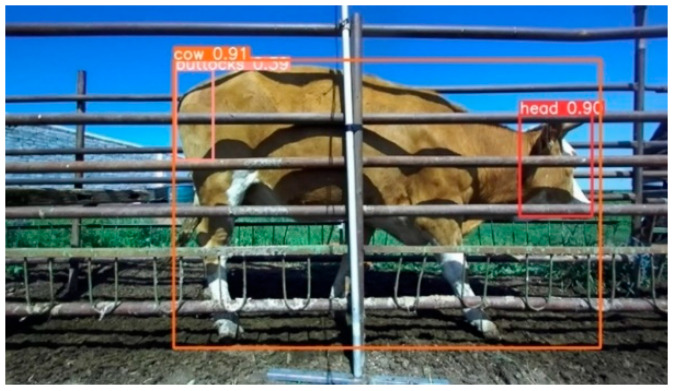
Actual scene YOLOv8 detects the critical region of the cattle body.

The single-view Zed2 camera embedded with the YOLOv8 target detection model was selected as the trigger controller for the automatic measurement device. This approach serves to shorten the installation time of the automatic measurement equipment and eliminate the risk of accidental triggering caused by physical triggers. The right camera embedded model with relatively optimal lighting conditions was selected to monitor the in-channel situation in real time and to specify the center of the field of view as the optimal shooting area. When the cattle are completely in the optimal shooting area, as in Figure 3, RGB images, depth images, and point cloud data are captured simultaneously in three views and grouped according to the cattle’s tag number.

### 2.2. Point Cloud Pre-Processing

Because the initial point cloud contains non-targeted point cloud areas outside of the cattle body, the raw data needs to be preprocessed. This ensures that subsequent point cloud alignment tasks can be completed.

**Figure 4:**
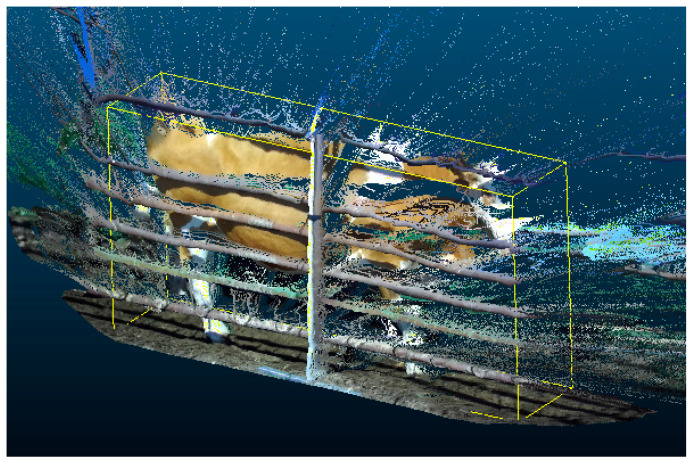
The yellow rectangular frame is used to initially crop the local cattle body point cloud from the original point cloud.

Straight-through filtering is applied to the multi-view point cloud to crop out the target area where the cattle body is located. The relative positions of the channel and the automatic measuring device are fixed, and the initial point cloud obtained by the same shooting logic determines the constant cropping area. A group of multi-view point clouds is randomly selected, and suitable boundary points are set as diagonal points of the rectangular enclosing frame of the straight pass filter. The rectangular enclosing box for the right views is shown in Figure 4. Each group of point clouds for different cattle applies the defined boundary points to eliminate the point clouds within the non-target area and initially crop the filtered point clouds.

**Figure 5:**
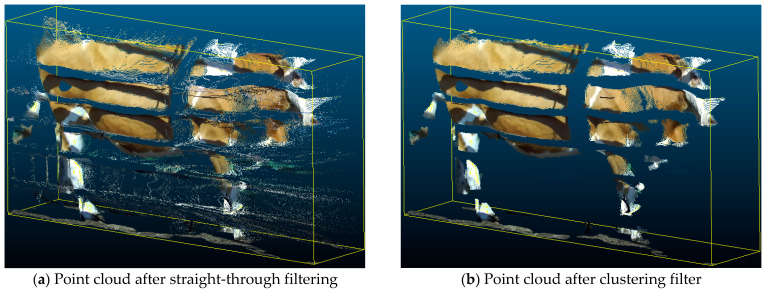
Comparison of local cattle body point clouds before and after clustering filtering. (**a**) Before clustering filtering. (**b**) After clustering filtering.

After straight-through filtering, the point cloud still contains randomly distributed clumps of environmental noise, as shown in Figure 5a. In order to improve the quality of the point cloud, cluster filtering and statistical filtering are applied to the further filtering process. Clustering filtering is implemented through the density-based DBSCAN algorithm. The target clusters that match the shape of the cattle body are identified and retained to remove the environmental noise.

For any point $p_{i}$ on the point cloud D, let the set of all neighboring point $p_{j}$ within the neighborhood r be denoted as:

(1) $$N_{r}(p_{i})=\{p_{j}:\left\|p_{j}-p_{i}\right\|<r\}$$

where $\left\|p_{j}-p_{i}\right\|$ represents the Euclidean distance between $p_{i}$ and any $p_{j}$ belonging to the neighborhood of $p_{i}$.

Set the minimum sample sizes N. Traverse each $p_{i}$ within D. When $N_{r}(p_{i})\geq N$, this point is considered as the core point. Based on these core points, spatially neighboring point clouds are aggregated into multiple clusters C. The mathematical expression is as follows:

(2) $$DBSCAN(D,r,N)=\{C|\forall p,q\in C,\left|N_{r}(p_{i})\geq N\right|\}$$

Analyze the morphological characteristics and sizes of the clusters. Filter out non-target clusters that do not match the shape features of cattle bodies. The influence of environmental noise is further reduced, as shown in Figure 5b.

Within the target cluster, there are still anomalous noise points that need to be cleaned up by statistical filtering. For each point $p_{i}$ within the target cluster, the mean distance $\mu_{i}$ and standard deviation $\sigma_{i}$ from $p_{i}$ to its k nearest neighboring point $p_{j}$ are calculated. The calculation formulas are as follows:

(3) $$\mu_{i}=\frac{1}{K}\sum_{j=1}^{K}\left\|p_{i}-p_{j}\right\|$$

(4) $$\sigma_{i}=\sqrt{\frac{1}{k}{\sum_{j=1}^{k}\left(\left\|p_{i}-p_{j}\right\|-\mu_{i}\right)}^{2}}$$

The mean distance $\mu_{i}$ represents the average dispersion between $p_{i}$ and its neighboring points. The standard deviation $\sigma_{i}$ measures the variation in these distance values.

The formula for identifying outliers $p_{d}$ is defined as:

(5) $$p_{d}=\{p_{i}:\left\|p_{j}-p_{i}\right\|>\mu_{i}+\alpha \sigma_{i}\}$$

Modifies the ideal sensitivity parameter α that is applicable to the point clouds of the various views. Identifies and removes apparently useless outlier noise in sparse regions at the edges of the point cloud and ensures that the largest number of valid data points (points that are close to the average distance of the surrounding points) are retained. It is ensured that subsequent tasks are minimally interfered with by these outlier noises.

After undergoing the rigorous processing flow described above, precise and high-quality multi-view point clouds are achieved. These point clouds serve as the solid foundation and reliable support for subsequent point cloud alignment tasks.

### 2.3. Multi-View Point Cloud Alignment

#### 2.3.1. Coarse Alignment

Since each view’s point clouds are gathered from various locations and perspectives, they each have their own unique spatial coordinate system. In order to create a complete and coherent point cloud model of the cattle body by combining these point clouds from various views. The first step is to carry out a coarse alignment to make sure that every point cloud can be initially aligned under a consistent spatial coordinate system. Because the top view is less impacted by the light and is not obscured by the fence. In this study, the point cloud of the top view is chosen as the reference point cloud of each group of point clouds, and the spatial coordinate system in which it is located is determined as the reference spatial coordinate system.

**Figure 6:**
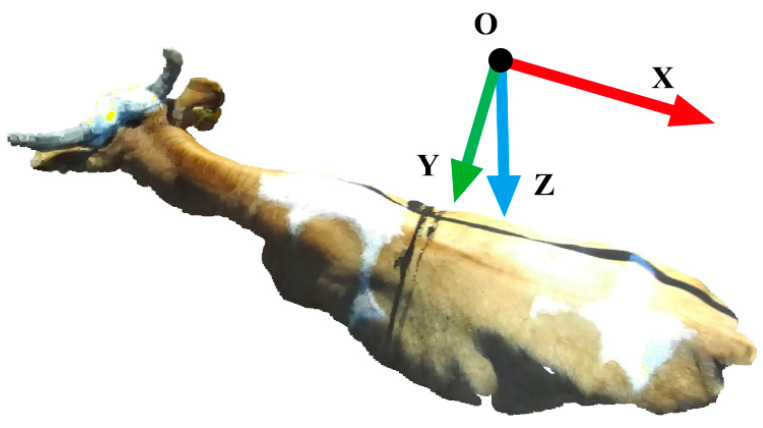
The reference spatial coordinate system.

The reference spatial coordinate system takes the top zed2 camera location as the origin; the direction of the right eye of the camera is the direction of the x-axis, pointing to the cow’s hip; the y-axis is coplanar and perpendicular to the x-axis, pointing in the direction of the left view; the z-axis is pointing to the ground, as shown in Figure 6.

Subsequently, the point clouds of the left and right views are approximately aligned with the datum point cloud in the reference spatial coordinate system by rotation and translation transformations. The rotational translation of the left and right views can be represented by 4 × 4 transformation matrices $\left(T_{i},i=1,2\right)$, and each change matrix $T_{i}$ consists of the 3 × 3 rotation matrix $R_{i}$ and the translation vector $t_{i}=\left(x_{i},y_{i},z_{i}\right)\;$:

(6) $$T_{i}=\left(\begin{array}{cccc} & R_{i} & & {\left(t_{i}\right)}^{T}\\ 0 & 0 & 0 & 1 \end{array}\right)$$

where the rotation matrix $R_{i}$ is derived from the angular difference in field of view positions between the left and right view cameras and the top camera. The translation vector $t_{i}$ is approximated by the relative positions of the left and right cameras and the top camera mounted in the reference spatial coordinate system.

**Figure 7:**
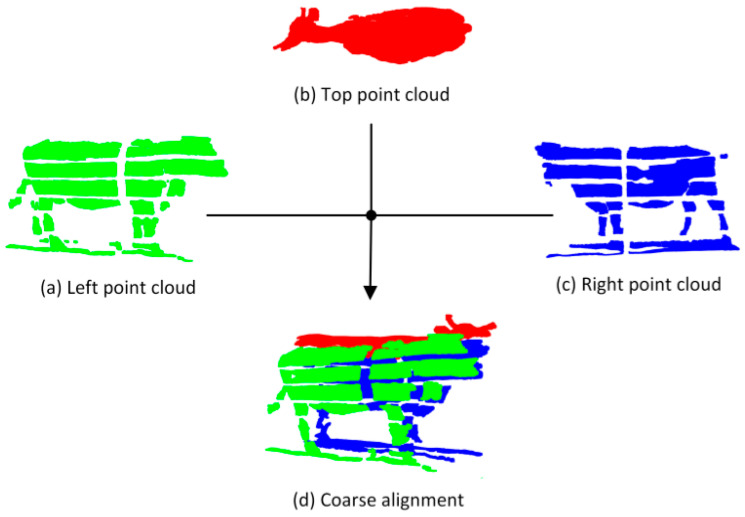
Point clouds at different views are roughly aligned to the point cloud of the cattle body after coarse alignment.

Figure 7 illustrates how the transformation matrix $\left(T_{i},i=1,2\right)$ generally aligns the point clouds of the right and left views with the top view point cloud in the reference spatial coordinate system.

#### 2.3.2. Fine Alignment

The point cloud model of the cattle body still has discontinuities even after coarse alignment with the transformation matrix $\left(T_{i}^{j},i=1,2\right)$. Light-induced point cloud distortions as well as point cloud voids due to railing occlusions can affect local features calculated based on the spatial distribution of neighboring points, as in Intrinsic Shape Signatures (ISS3D) [19]. This can lead to a loss of local feature consistency at points that should correspond to each other in different views, which makes it impossible to realize the provision of accurate alignment information. The Iterative Nearest Point (ICP) algorithm, which does not depend on the spatial distribution of the neighborhood, enables fine alignment of point clouds. The ICP algorithm achieves rigid point cloud alignment by iteratively minimizing the Euclidean distance of the corresponding points between the source and destination point clouds.

**Figure 8:**
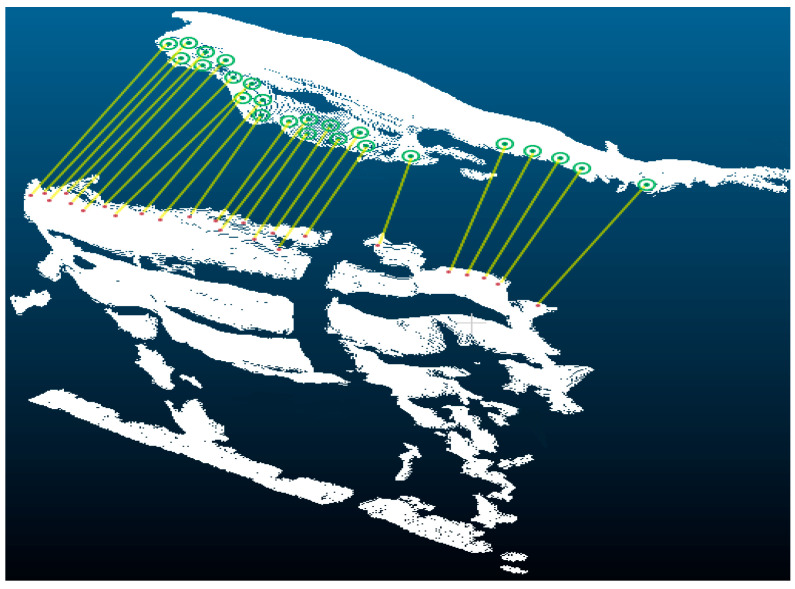
The right view point cloud is used as the source point cloud and the top view point cloud is used as the target point cloud. The source point cloud is made close to the target point cloud by iterative transformation.

However, the more robust point-to-plane ICP algorithm is used in this study. The point-to-plane ICP algorithm projects the points in the source point cloud onto the faces of the target point cloud by adding normal vector information. The point-to-plane distance is decreased to achieve more accurate non-rigid point cloud alignment, as shown in Figure 8. Compared to the conventional ICP algorithm, it is better suited for non-rigid point cloud alignment tasks like cattle bodies [23].

The point cloud that will be used as a reference is marked as the target point cloud. The initial point cloud that needs to be transformed to align with the target point cloud is marked as the source point cloud. The specific steps of the point-to-plane ICP algorithm are as follows:

For each point in the source point cloud, find its corresponding nearest point in the target point cloud. Determine the local normal direction at that nearest point;

Compute the optimal transformation based on the point-to-plane distance metric. Minimize the average distance from all points in the transformed source point cloud to the corresponding local plane;

Apply the computed transformation to the source point cloud to update the position of the source point cloud;

Repeat steps 1–3 until the convergence criterion is satisfied. The change in the transformation is less than the set threshold or the predetermined number of iterations is reached.

Due to the large size of the cattle and the limited field of view of the camera, the point clouds acquired from different views have a low overlap with each other. However, the ICP alignment requires a large overlapping area between the point clouds. It is difficult to obtain stable alignment results by directly using the point-to-plane ICP algorithm for alignment [24].

The presence of non-overlapping regions in the point clouds results in inaccurate corresponding point relationships derived from different views. These regions not only reduce the alignment efficiency but also lead to alignment inaccuracy. Therefore, the alignment possible region $P_{overlap}$ is computed to solve this problem: Using the relative positions between the source and target point clouds after coarse alignment, the region of a high probability overlap between the two point clouds is calculated.

Point clouds after coarse alignment are denoted as the query point cloud $P_{query}$ and the model point cloud $P_{model}$. For all k points $D_{q}$ in the $P_{query}$ after searching their i nearest neighbors $D_{m}$ in the $P_{model}$ using the KD tree, the distance dis(k) between them is calculated as:

(7) $$dis(k)=\min\left\|D_{q}(k)-D_{m}(i)\right\|,k,i=1,2,3\cdots \cdots$$

The smallest of these distances dis is chosen as the description of the distance from the $D_{q}$ to the $P_{model}$.

Due to the large amount of point cloud data, the computational complexity of directly calculating the nearest distance from the points in each $P_{query}$ to the $P_{model}$ is high, which leads to the increase in the alignment time. To alleviate this problem, the data structure based on Minimum Heap is used to optimize the computation process. The effect of the Min Heap is simulated indirectly by storing the negative distance −dis to efficiently filter out the nearest points to the $P_{model}$. The pseudocode for the specific steps is shown in Appendix A Algorithm A1.

**Figure 9:**
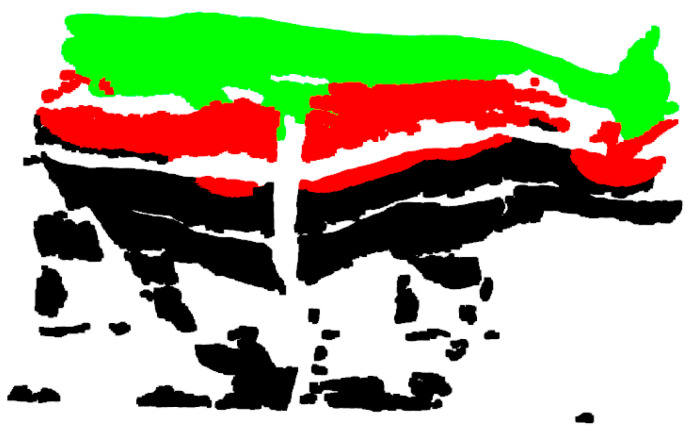
The green area is the top view point cloud being used as the $P_{model}$. The red area is the $P_{overlap}$ in the right view point cloud.

The final heap stores the nearest distance dis from the $P_{query}$ to the $P_{model}$ and its corresponding index. The number of elements is $\left\lfloor P_{query}\right.\left.\times f_{p}\right\rfloor$. The indexes in the $P_{query}$ corresponding point, that is the $P_{overlap}$, satisfy $P_{overlap}\subseteq P_{query}$. The computed $P_{overlap}$ with $f_{p}$ set to 25% is shown in Figure 9. A low $f_{p}$ value could lead to insufficient information and jeopardize the stability of the alignment. On the other hand, an excessively large $f_{p}$ may result in an excessive amount of noise and mis-correspondence points, which would lower the alignment’s accuracy.

The $P_{overlap}$ is used as the source point cloud. The $P_{model}$ is used as the target point cloud. Execute the point-to-plane ICP algorithm. After excluding interference in non-overlapping regions, the point-to-plane ICP algorithm is able to operate in smaller and more correlated regions. This reduces the amount of computation. Meanwhile, the accuracy of the correspondence between the source and target point clouds is improved, thus enhancing the robustness and accuracy of the point-to-plane ICP algorithm alignment.

In this study, the point cloud captured from the top view, characterized by unobstructed and relatively uniform illumination, was chosen as the $P_{model}$. The point clouds from the left and right views were utilized as $P_{query}$ to compute the $P_{overlap}$. Next, the point cloud of the vertex view is used as the target point cloud for the point-to-plane ICP algorithm. The point clouds of the right and left views are used as source point clouds for iterative computation of the optimal fine alignment transformation matrix. Ultimately, the multi-view point clouds, which were initially coarsely aligned, undergo further alignment using two finely transformed matrices.

**Figure 10:**
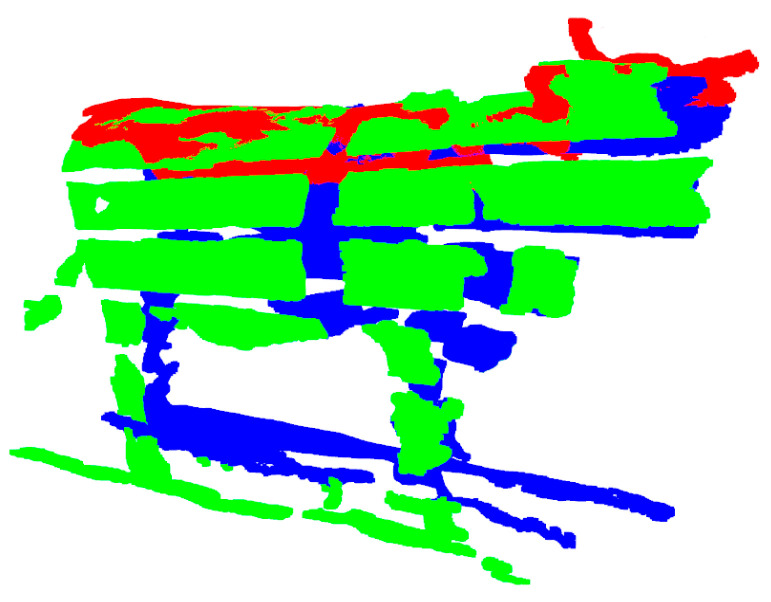
The complete and coherent point cloud model of the cattle body.

As shown in Figure 10, a comprehensive and coherent, 3D model of the cattle body was produced, consisting of a point cloud. Its spatial coordinate system is consistent with the point cloud of the top view. Subsequent body size measurements were taken in conjunction with the 3D cattle body model.

### 2.4. Measure Cattle Body Sizes

#### 2.4.1. Cattle Body Key Point

The anatomical structure of the cattle body varies significantly depending on its breed, age, sex, and body condition. It is inaccurate to use the anatomical structure information directly to locate the key points. The YOLOv8x-pose key point detection algorithm was used to obtain key point information on 2D RGB images. The key points were mapped onto the 3D cattle body for subsequent body size measurements. The YOLOv8x-pose model for detecting the key points of the cattle body is trained for 1200 sets of RGB images of cattle exhibiting various postures in the channel.

**Figure 11:**
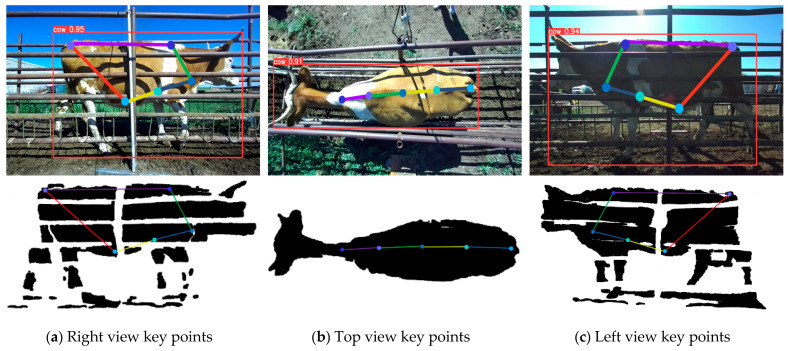
Key points detected by YOLOv8x-pose in three views. Mapped key points on the point cloud for each view. (**a**) The key points for the right views were consistent and were: dorsal fins highest point (back), sciatic end (buttocks), shoulder end (foreleg), abdominal widest point (abdomen), and scapular posterior corner of the scapulae (chest); (**b**) For the top views, the key points were: neck (neck), dorsal fins highest point (back), abdominal widest point (abdomen), auxiliary point (aid), and sciatic end (buttocks); (**c**) The left views mirror the same key points as the right views.

As shown in Figure 11, the pixel coordinates (x,y) of the key points of the cattle body are obtained in the RGB image of each view separately. Then, the 2D key points are mapped onto the 3D cattle body by combining the depth value z and the camera internal reference. The calculation formula is as follows:

(8) $$\left\{\begin{array}{l} X=\frac{(x-c_{x})z}{f_{x}}\\ Y=\frac{(y-c_{y})z}{f_{y}}\\ Z=z \end{array}\right.$$

where (X,Y,Z), are the coordinates of key points in the point cloud, also known as 3D key points. When the camera is turned on, internal parameters such as camera focal length $f_{x}$, $f_{y}$ and camera optical center coordinates $c_{x}$, $c_{y}$ are adjusted adaptively according to the actual environment.

#### 2.4.2. Measure Linear Body Sizes

Railings do not occlude the point cloud from the top view. Five key points (neck, back, abdomen, aid, and buttocks) are accurate tracking of the dynamics of the cow spine that closely monitor how the cattle body’s spine line changes in various positions. They guarantee that the cattle in various postures can have their body straight lengths $L_{s}$ precisely estimated.

The Euclidean distance $L_{ij}$ between two neighboring points was calculated for each of the five key points of the top views from the neck key point to the buttocks key point. Accumulation of these distances gives the approximation of the body straight length $L_{s}$, which was mathematically expressed as:

(9) $$L_{ij}=\sqrt{{\left(x_{j}-x_{i}\right)}^{2}+{\left(y_{j}-y_{i}\right)}^{2}+{\left(z_{j}-z_{i}\right)}^{2}}$$

(10) $$L_{s}=L_{12}+L_{23}+L_{34}+L_{45}$$

The z-axis coordinate of the ground is set to $H_{0}$. For each of the three views, the z-axis coordinates of the back key points are indicated by the symbols $z_{1}$, $z_{2}$, and $z_{3}$. The height difference between $H_{0}$ and the three is then computed.

In order to minimize the mistake brought on by cattle postures, the average of the three height discrepancies derived from multi-view measurements was utilized to estimate the estimated body height $L_{h}$. The approximate body height $L_{h}$:

(11) $$L_{h}=\frac{\left(z_{1}-H_{0})+(z_{2}-H_{0})+(z_{3}-H_{0}\right)}{3}$$

Although the back key points in both side views may be occluded by railing, causing the back key points to be projected onto the railing. However, this problem can be avoided if only z-axis coordinate values are used. This is because even if the projected point is on the railing, it has the same z-axis coordinate value as the ideal back key points.

The buttocks key point and foreleg key point in the right and left views were used as measurement key points. The Euclidean distance between the two points was calculated as the body slant length $L_{ol}$ and $L_{or}$. The average of the two views was used as the final approximation of the body slant length $L_{o}$:

(12) $$L_{o}=\frac{\left(L_{ol}+L_{or}\right)}{2}$$

Similarly, both the buttocks key point and the foreleg key point may be obscured by the railing, causing the key point to be projected onto the railing. The direct use of the Euclidean distance between the two important sites as the body slant length $L_{ol}$ and $L_{or}$, must be abandoned in the event of occlusion. It is necessary to project the two points onto the y = 0 plane first to compute the 2D plane distance as $L_{ol}$ and $L_{or}$.

#### 2.4.3. Measure Circumference Body Sizes

**Figure 12:**
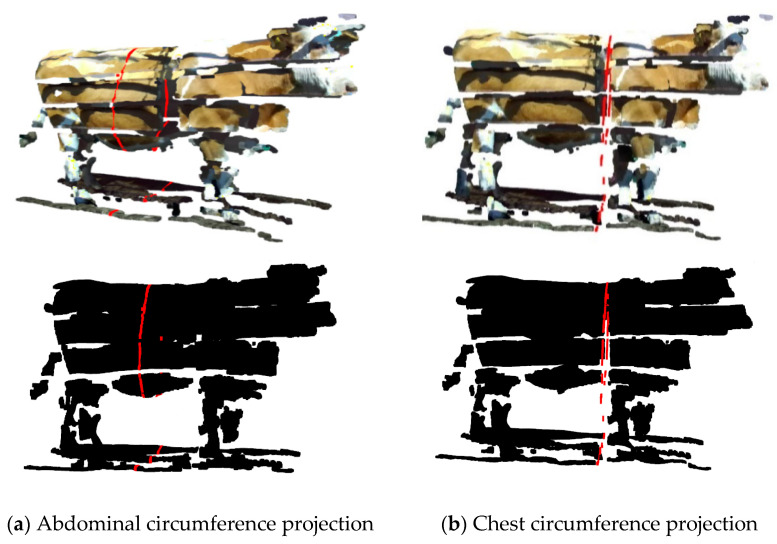
Spatial location of the abdominal and thoracic projection planes.

When obtaining the circumference projection, the reference point is first defined. For the abdominal circumference, the abdomen key point in the side view is chosen as the reference point. For chest circumference, the chest key point is chosen as the reference point. Based on the reference point, the plane normal vector is defined to determine the spatial location of the circumference projection plane, as shown in Figure 12. If the railing obscures these two key points, it does not prevent obtaining the correct plane of projection. As long as the selected reference point and the ideal key points are on the same projection plane, the subsequent measurements will not be affected. Even if the abdomen key point and chest key point are located on the railing, they still satisfy the requirement. Finally, the point cloud within the tolerance range is projected onto this plane to obtain the circumference projection to be measured. The tolerance range accounts for any circumference data that might be obscured by the railing, ensuring that sufficient data are projected onto the plane for measurement.

**Figure 13:**
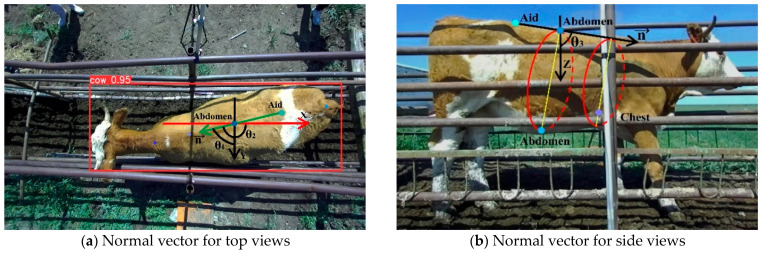
Plane normal vectors and circumference projection planes in non-standard postures. The vector n pointing from the aid key point to the abdomen key point at the top views is chosen as the normal vector of the circumference projection plane. Its angles to the yOz, xOz, xOy planes are $\theta_{1}$, $\theta_{2}$, $\theta_{3}$.

Manual measurements under the current standards are then generally carried out in the standard stance. However, the standing posture of livestock in the channel is divided into standard and non-standard postures [25]. The use of the constant normal vector does not allow stable acquisition of the plane of the enclosure projection in the standard stance. Therefore, each time the circumference projection is acquired, the independent projection plane normal vector needs to be computed for each group of point clouds to normalize the stance of the cattle. Two key points on the flattest outer ridge portion of the cattle’s back in the standard stance were selected: the abdomen key point and the aid key point in top views. Calculate the normal vector of the projection plane shown in Figure 13.

This ensures that each group of point clouds acquires the plane of circumference projection at the current attitude that is angularly similar to the standard attitude. The circumference projection data with similar distribution properties is acquired. After applying the percentage threshold to exclude extraneous data such as ground. The final circumference projection data exhibits ellipse-like distribution properties in the Cartesian coordinate system.

**Figure 14:**
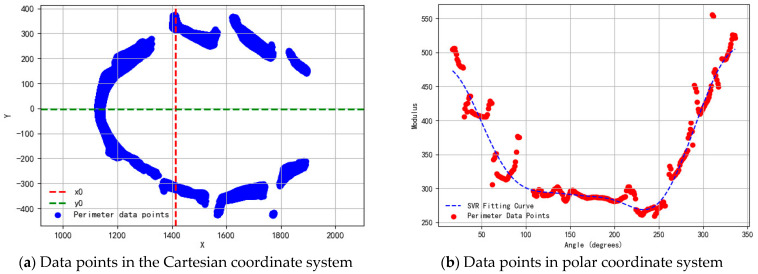
Distribution of data points in the projection plane of circumference. (**a**) The point $\left(x_{0},y_{0}\right)$ in the original data is selected as the origin of the polar coordinate transformation; (**b**) The fitted curves of SVR always capture the main trends of the raw data.

However, the circumference projections were missing and deformed in the localized range due to the influence of environmental factors such as railings obscuring the cattle and uneven natural lighting. The support vector regression (SVR) model, a type of supervised learning algorithm used for regression analysis, was used to fit and repair the affected circumference profiles [26]. To optimize computational efficiency, the origin of the point $\left(x_{0},y_{0}\right)$, which is inside the elliptical data, was selected as the origin to transform the Cartesian coordinate system to the polar coordinate system, as shown in Figure 14. (**a**) The point $\left(x_{0},y_{0}\right)$ in the original data is selected as the origin of the polar coordinate transformation; (**b**) The fitted curves of SVR always capture the main trends of the raw data. The data points are represented by the polar diameter ρ and the polar angle θ:

(13) $$\theta =\frac{\pi }{180}\times (\mathrm{atan}2(y-y_{0},x-x_{0})+\pi )\mathrm{mod}360$$

(14) $$\rho =\sqrt{{\left(x-x_{0}\right)}^{2}+{\left(y-y_{0}\right)}^{2}}$$

where $\theta \epsilon [0^{{}^{\circ}},360^{{}^{\circ}}]$ is used as the new x-axis. The corresponding polar diameter ρ is used as the new y-axis.

Through this transformation, data points that originally showed complex distribution in the Cartesian coordinate system become simple distributions in the polar coordinate system. This helps to improve the efficiency and accuracy of the SVR model in predicting missing data, as shown in Figure 14.

After defining the range of the parameter space distribution of SVR, random search cross-validation is used to explore the parameter space. The optimal SVR model parameters are found with the goal of minimizing the mean square error. The SVR model is then instantiated using the best parameters. After fitting the repair circumference data in fitted polar coordinates, it is transferred back to the Cartesian coordinate system to obtain the initial repair circumference profile.

**Figure 15:**
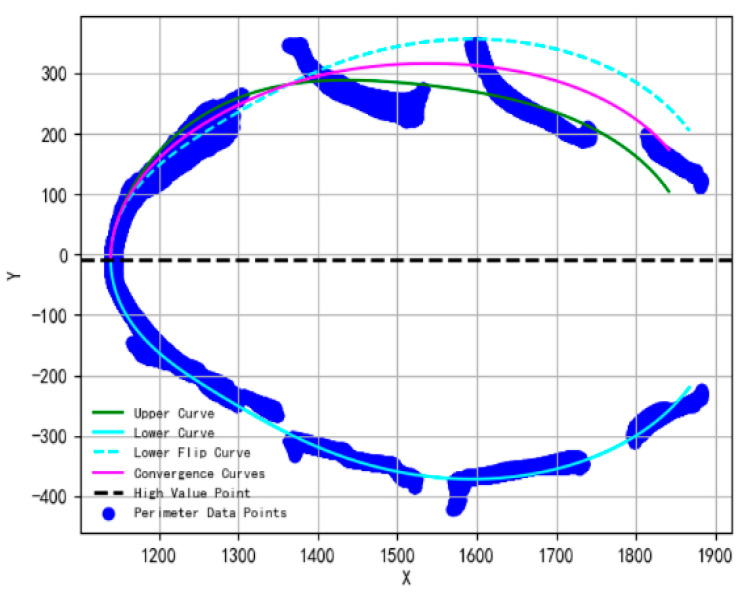
The new upper curve after fusion. Resists the original inward deformation well shape-wise.

The fitted curves were divided into upper and lower sections (right and left views) along the y-axis of the maximum point. Since the upper section of the curve corresponding to the left views is the data collected at the backlight, the degree of missing deformation is serious, and the prediction accuracy is relatively low. The body structure of quadrupeds such as cattle tends to show approximate symmetry on the left and right sides [27]. The distribution of the circumference data points tends to be consistent after posture normalization. The circumference fitting curve is always approximately symmetrical between the upper and lower parts. Therefore, curve fusion can be used to improve the accuracy of the variation in the fitted curves. Specifically, the lower section of the curve (right views), which has a relatively small degree of deformation, is flipped and aligned with the upper segment curve (left views). The mean values of the corresponding points of the two curves are calculated to achieve a curve fusion. After this process, the influence of deformation is reduced; the new upper curve is smoother in shape. This is shown in Figure 15.

Due to camera angle limitations and oversized cattle, the circumference projection data were completely missing within the bottom area of the cattle. It was not possible to achieve the accurate prediction of this part directly in the available data through SVR.

**Figure 16:**
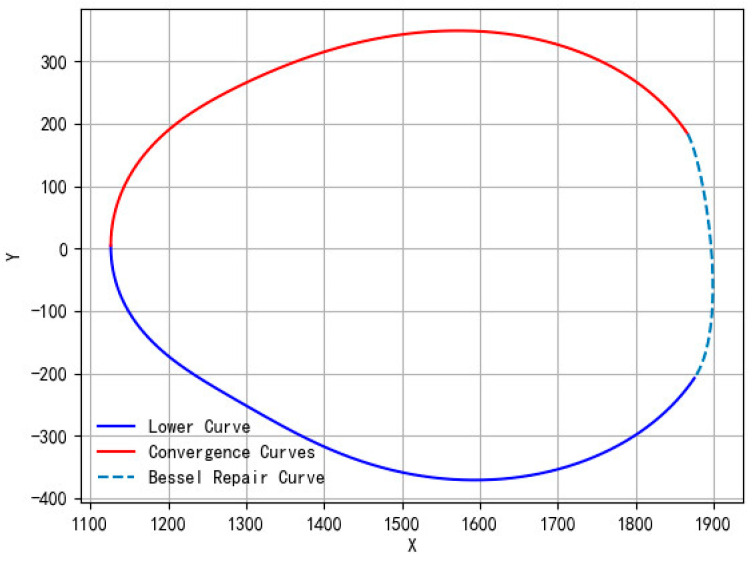
The complete abdominal circumference curve consists of three parts: the new upper curve after fusion, the SVR lower segment curve, and the Bessel repair curve.

In order to maintain the integrity of the fitted curves for circumference, a smooth connection technique based on local geometric feature analysis was used to fill in the missing parts. In the preliminary fitted upper and lower curves of SVR, the breakpoints with the largest values of their respective x-axis coordinates were selected as the points to be connected, denoted as $P_{0}$ and $P_{1}$. The local trends of the fitted curves in the vicinity of the to-be-connected points were captured by calculating the slopes of the tangents of $P_{0}$ and $P_{1}$ to the neighboring points. The optimal control points $P_{c1}$, $P_{c2}$ of the cubic Bessel curve in the upper and lower curves are deduced. The parametric equation of the cubic Bessel curve is expressed as:

(15) $$B(t)={\left(1-t\right)}^{3}P_{0}+3{\left(1-t\right)}^{2}tP_{c1}+3(1-t)t^{2}P_{c2}+t^{3}P_{1}$$

where tϵ[0,1]. Varying the value of t generates a series of points with smooth transitions as connection points for the repair curves. This ensures that both the repair curve and the fitted curve can be seamlessly connected at the breakpoints $P_{0}$ and $P_{1}$, and there are no abrupt jumps or breakpoints in the repair curve. The final complete circumference curve is shown in Figure 16. The sum of the perimeters of the three curves is the corresponding circumference body sizes.

## 3. Results

This study, was in collaboration with the cattle ranch in Wulagai Management Area, Xilingol League, Inner Mongolia. The automatic measurement device was deployed outdoors in the natural light environment on the channel leading from the pen to the pasture for the cattle. In this study, a total of 47 Huaxi beef cattle of different ages passed through the measuring device, covering a wide range of coat colors and body sizes. This ensures that the samples are representative of a wide range of characteristics of the breed, thus increasing the reliability of the validation of the measurement method; the stance of the cattle as they enter the measurement device is random, without human intervention, and has randomness and diversity.

When the cattle are walking to pasture in the channel and reach the designated capture area monitored by the YOLOv8, the multi-view camera automatically acquired the RGB images, depth images, and point clouds at the same time. Four body sizes were then automatically measured: linear body sizes including body height (BH) and body length (BL), and circumference body sizes including abdominal circumference (AC) and chest circumference (CC). After passing through the equipment, the cattle were then manually measured by two experts using a tape measure for each of the four body size data. The final manual measurements were determined by averaging the measurements taken by the two experts. In this case, circumference body sizes were measured for all cattle, and linear body sizes were measured for 12 of them, selected randomly.

### 3.1. Circumference Data Fitting Restoration

Because of occlusion and illumination, there were many missing and outliers in the original circumference data. The data were repaired using the support vector regression machine (SVR) technique. The mean absolute percentage error (MAEP) and the average of the coefficients of determination ($R^{2}$) were used to measure the degree of model fit. The better the model captures the data’s trend, the closer the $R^{2}$ number is to 1; the smaller the MAEP value, the less the model-data error. The following are the precise formulas:

(16) $$R^{2}=1-\frac{\sum_{i=1}^{n}{\left({\hat{y}}_{i}-\bar{y}\right)}^{2}}{\sum_{i=1}^{n}{\left(y_{i}-\bar{y}\right)}^{2}}$$

(17) $$MAEP=\frac{1}{N}\sum_{i=1}^{N}\left|\frac{y_{i}-{\hat{y}}_{i}}{y_{i}}\right|\times 100\%$$

where $y_{i}$ is the actual observation, ${\hat{y}}_{i}$ is the prediction of the model, $\bar{y}$ is the mean of all observations, and N is the sample size.

Table 1 displays the outcomes of the parametric assessment of the SVR-fitted circumference curves. By closely following the original data’s trend, SVR is able to match the missing data for both AC and CC. No significant extra errors are produced.

However, because of the influence of light, the upper section of the SVR-fitted curve (left view) may exhibit considerable erroneous distortion. The average of the smoothing metrics (smoothness) is presented to measure the impact of this distortion. The smoothness of the data sequence indicates the lack of significant fluctuations or sudden shifts; the closer the smoothness is to 0, the smoother it is. The following is the precise calculation formula:

(18) $$smoothness=\frac{1}{n-1}\sum_{i=1}^{n-1}\left|y_{i+1}-y_{i}\right|$$

Table 2 shows how the fitted curves’ smoothness changed for the upper section of the SVR at the same number of points before and after curve fusion. The curve fusion technique greatly improves the original poor smoothness, resulting in more accurate and smooth fitted curves.

### 3.2. Comparison of Automatic and Manual Measurements

**Figure 17:**
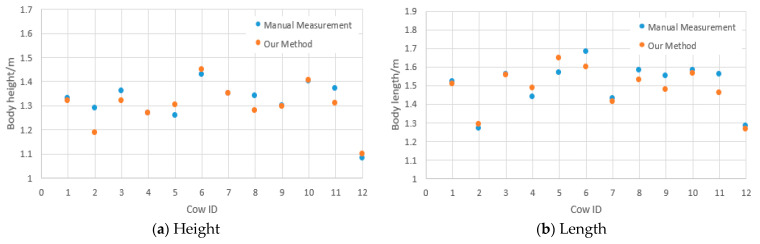

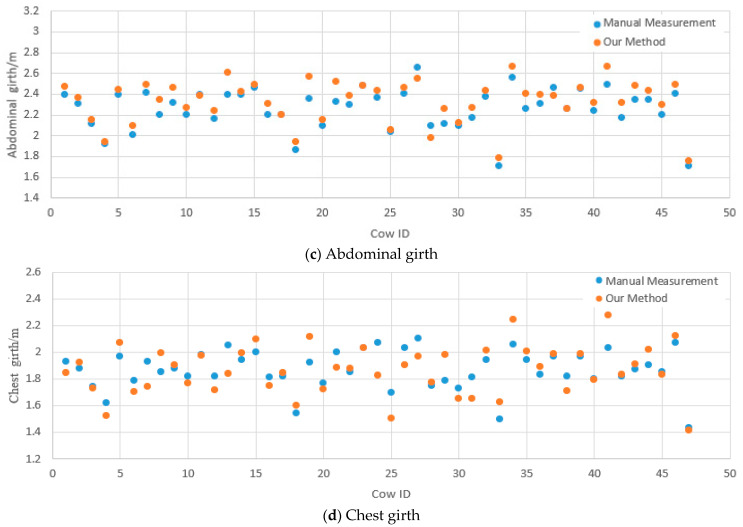
Four body size parameters: body length, body height, abdominal, and chest circumference. Comparison of manual and automated measurements.

In order to analyze the accuracy of the automatic measurement method of body size parameters in this study, the manual measurements at the time of data acquisition were used as the reference to evaluate the results, as shown in Figure 17.

BH indicates skeleton size and growth stage, impacting feed formulation and density, ensuring proper nutrition and growth. BL provides insights into body size and structure, aiding weight estimation, and breeding plans. AC reflects muscle and fat content, which is crucial for illness prevention, treatment, and monitoring nutritional health. CC assesses body condition and respiratory function, which is crucial for health and economic value. They collectively form the basis for cattle health evaluation and feeding management.

The comparison of manual measurement of MAE and MRE for the four body sizes is summarized in Table 3. The MREs for all four body categories satisfies the requirements of ranchers who wish to measure the body sizes of cattle inside the 8% MRE.

The error in the chest girth is the largest. The thoracic region of the cattle exhibits a steeper contour change on the left and right sides compared to the abdominal region. This results in significantly fewer point clouds in the chest region than in the abdomen region from different views. This uneven distribution of point clouds makes the point cloud alignment process more inclined to enhance the accuracy of the abdominal region and neglect the chest region. This results in a larger error in the measurement of the chest circumference compared to the abdominal circumference.

**Figure 18:**
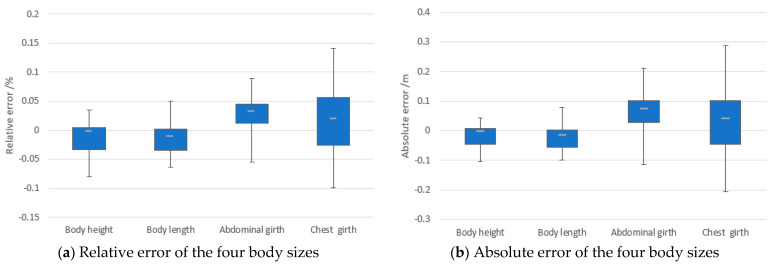
The box plot of the four-term body sizes. The center line inside the box represents the median, and the whiskers outside the box are the maximum and minimum values.

The box plots in Figure 18 visualize the error distribution between manual and automatic measurements, and statistically, the overall measurements have the mean relative errors (MRE) of 3.50% and the mean absolute error (MAE) of 6.33 cm. Among them, the MRE of body height was 2.32%, and the absolute error control was −10.4–4.4 cm; the MRE of body length was 2.27%, and the absolute error control was −10.0–7.8 cm; the MRE of abdominal circumference was 3.67%, and the absolute error control was −11.4–21.2 cm; the MRE of chest circumference was 5.22%, and the absolute error control was −20.5–28.6 cm.

## 4. Discussion

### 4.1. Applications and Challenges of Automatic Measurement in Range Management

Since physical cattle indicators are closely linked to a herd’s health, productivity, and general operational efficiency, ranch administrators place a lot of focus on keeping an eye on these indications. Hand restraint, chute restraint, and chemical restraint are examples of traditional manual measurement techniques that, although they partially meet fundamental measurement requirements, have serious drawbacks.

Despite being straightforward and simple to use, the hand restraint approach is only appropriate for smaller or more submissive calves. In addition to being ineffectual, it presents serious safety hazards for larger or more temperamental cattle, possibly causing stress reactions that negatively impact the animals’ physical and emotional health. Although chemical restraint temporarily incapacitates the cattle, it can also result in health problems, has a short duration, and necessitates precise dosage and timing control, which increases the operation’s complexity and risk. As the most common approach now in use, chute restraint improves measuring accuracy and safety to some extent but requires significant space and equipment investments in addition to laborious and time-consuming processes. It also negatively affects the cattle’s welfare by putting them through needless stress and constraint.

As a result, the introduction of non-contact autonomous measurement technology is a revolutionary change for ranch management, providing a host of unique benefits like avoiding stress reactions and removing safety dangers, among others. This technology has become the mainstream trend in future pasture management.

Channels for chute restraint are mostly built outdoors, connecting the cattle barn to the pasture. To prevent calves from escaping, the channels are often constructed with dense, thick railings. This study’s automatic measurement device leverages this existing outdoor pathway.

However, practical applications necessitate overcoming multiple interferences, including uneven outdoor lighting and railing occlusion. Extreme variations in lighting intensity can lead to significant issues: excessively high light levels can cause images to be overexposed, a problem that is exacerbated on sunny days, while excessively low light levels can result in underexposure, which is more pronounced on cloudy days. Overexposure not only produces small holes and white blocks, but also outlier noise; underexposure also produces outlier noise, as well as reduces contrast and exacerbates the effects of the railing, leading to distorted and warped point clouds in the vicinity of the railing. These phenomena pose a threat to the accuracy and integrity of the point cloud.

To address these challenges, a series of innovative methods and technical approaches have been adopted. Firstly, multiple filtering techniques were employed to eliminate the influence of isolated points and white blocks. Secondly, to address the issue of point cloud feature from different views correspondences due to holes and distortions, which prevent direct use of point cloud feature alignment, the point-to-plane ICP algorithm combined with the potential alignment region technique was used to obtain stable alignment results, effectively solving this problem. Lastly, methods such as support vector fitting, curve fusion, and Bézier curves were utilized to effectively repair the holes and distortions affecting the circumference body size measurements.

### 4.2. Comparing Automatic Measurement in Different Studies

There are two main types of multi-view automated measurements: channel and non-channel. Non-channel methods, such as Yang et al. [28], still require violent confinement of livestock, which does not improve animal welfare and may still pose a safety hazard; channel methods do not require violent confinement of livestock, but previous studies have been conducted in specialized scenarios with uniform indoor lighting and fewer, thinner railings. In this study, automated measurements were performed on an existing outdoor channel made of thick and dense railings. There was no need for more location, equipment, or labor. In addition, there is no way to adapt to the different postures of cattle to measure the body size in Shuai et al. [29]—only a single posture can be measured. The use of key points to achieve the posture normalization approach in this study is more objective and efficient compared to the use of body curves on both sides to calculate posture in the study Hao et al. [30].

In Table 4, MRE regarding the automatic measurement of body size parameters from different studies are compared. Compared to most studies conducted indoors in controlled environments, it maintained high accuracy under various livestock postures, with a slight 1.01% increase in average MRE compared to previous best indoor results. This small difference not only validates the validity and reliability of our automated measurement method under natural light conditions but also provides strong technical support for outdoor practice of livestock body size measurement. It has wide potential and significant advantages in practical applications.

### 4.3. Limitations and Future Work

Understanding the limitations encountered in the current experiment, where the fence stands a considerable distance from the measuring device and the automatic measurement device in the cattle’s line of sight causes stress, making them hesitant to proceed, it is imperative to address these challenges in future work. To mitigate the visual stress on cattle, future designs should emphasize more discreet measuring devices that blend seamlessly into the channel’s structure, thereby encouraging a more natural flow of cattle through the area. Additionally, recognizing the failure of the automatic measurement device when multiple cattle enter the channel simultaneously, efforts must be directed toward developing measuring devices or algorithms capable of adapting to such crowded conditions, ensuring accurate measurements across a broader range of applications.

Environmental factors that interfere with measurement accuracy are difficult to eliminate completely. The high density and large size of railings as well as uneven lighting can exacerbate point cloud distortion. Railings can mask information about overlapping regions of the point cloud arrangement in different viewing angles. The chest has a smaller number of point clouds and naturally lacks information about the overlapping regions. The reduction of overlapping point clouds leads to a decrease in the accuracy of point cloud alignment. If the cattle collide with a railing, the cattle point cloud becomes completely mixed with the point cloud of the railing. Subsequently, it will become very difficult to separate and extract the point cloud of the cattle body. In order to solve these problems, more advanced point cloud processing algorithms need to be developed to reduce the impact of factors such as railings on point cloud alignment accuracy.

## 5. Conclusions

This study proposes a method for the automatic measurement of body sizes of cattle with different postures suitable for the outdoor complex environment, aiming to solve the limitations of existing livestock body size measurement techniques in practical applications. This study combines multi-view RGB images, depth images, and point clouds. The combination of coarse and fine alignment is used to construct the 3D cattle body. Important body size parameters such as body length, body height, abdominal circumference, and chest circumference were measured by the 3D-mapped key points of the cattle body obtained by the YOLOv8x-pose key point detection algorithm. The SVR model and Bessel curve technique were used to repair the missing data and deformation to achieve high accuracy body size measurement.

Compared with previous studies, this study conducted experiments in outdoor, more complex environments, and the cattle were able to maintain a high measurement accuracy even in a non-standard posture. Our method obtained the average relative error (MRE) of 3.50% in the outdoor complex environment, which differed by only 1.01% from the optimal results of the study in the controlled indoor environment, validating its validity and reliability under natural light conditions. Overall, this study provides a new idea and technical support for the automatic measurement of livestock body sizes, which has the prospect of application in practical pasture management.

## Figures and Tables

**Table 1:** Evaluation of SVR fitted circumference curves.

Norm	R^2^	MAEP (%)
AC	0.91	2.77
CC	0.88	3.38

**Table 2:** Smoothness of curves before and after fusion.

Norm	Pre-Fusion	Post-Fusion
AC	0.109	0.046
CC	0.091	0.056

**Table 3:** Summarize the MAE and MRE of the actual measured values of each body size parameter.

Norm	BH	BL	AC	CC
MAE/cm	3.04	4.27	8.29	9.71
MRE/%	2.32	2.77	3.67	5.22

**Table 4:** Comparison of MRE for each body size parameter in different studies.

Study	Animal	No Violent Confine	Outdoor Natural Light	Different Posture	Railing Obstructs the View	Mean Relative Error (%)			
						BH	BL	AC	CC
Shuai et al. [29]	Pig	√	×	×	L	2.97	3.35	4.13	\
Hao et al. [30]	Pig	√	×	√	L	2.57	2.28	3.14	2.85
Yang et al. [28]	Cattle	×	×	√	LL	1.69	2.41	\	4.00
Our	Cattle	√	√	√	H	2.32	2.77	3.67	5.22

Three levels of channel railing obstruction of targets: none (LL), slight (L) and severe (H); ‘\’: the study did not directly measure the labeled body size.

## Data Availability

Data are contained within the article.

## Appendix A

**Algorithm A1. Optimize Point Cloud Registration**
Input: $P_{query}$: Query point cloud with multiple points $P_{model}$: Model point cloud with multiple points $f_{p}$: the percentage value to determine the capacity of the min heap Output: H: The min heap H with capacity $\left\lfloor P_{query}\right.\left.\times f_{p}\right\rfloor$ is used to store distances and indexes ·For each point $D_{q}$ in $P_{query}$ do dis = $CalculateNearestDistance(D_{q},P_{model})$ neg_dis = −dis If H.sizes() < $\left\lfloor P_{query}\right.\left.\times f_{p}\right\rfloor$ H.add(neg_dis, ${index\_of\_D}_{q}$) · ElseIf $neg_{dis}$ > H.peek() H.removeMin() H.add(neg_dis, ${index\_of\_D}_{q}$) End
